# Hospital Progress and Finance

**Published:** 1924-09

**Authors:** 


### HOSPITAL PROGRESS AND FINANCE.

### THE SCARCITY OF HOSPITAL BEDS.

Before the Local Voluntary Hospital Committees start upon the inquiry they have been asked to make into the number of beds available in the hospitals of England and Wales, they have had a useful conference with the Voluntary Hospitals Commission. It does not necessarily follow that because more beds are demanded the supply is as inadequate as might be suggested by the difference between the existing number and that which might be supposed to be needed. Much depends upon the use made of the beds and upon whether they are always occupied inevitably. As regards cases of surgical tuberculosis, for instance, it was suggested at the Conference by one speaker that a surgical wing should be added to every sanatorium, and by another that the Public Health authority should look after such cases. The latter would seem to be the simpler and cheaper course. But, as Lord Cave pointed out, sufficient hospital accommodation means much more than a sufficiency of beds, and it is this larger question which will have to be met and solved.

---

### SHOULD A HOSPITAL TELL?

A recent decision in the Edinburgh courts has laid it down that a hospital must produce its medical records when a case turns upon medical evidence. That, at least, was the effect of Lord Ashmore's refusal to uphold the Edinburgh Sick Children's Hospital, which had objected to produce its records in connection with a claim for damages. It is affirmed that, in this particular case, the rights of the patient were not infringed, and without all the details before us it is impossible to determine whether the hospital chose its ground wisely. With the general principle that it endeavoured to defend it is easier to sympathise, for are not the relations of doctor and patient the same in institutional and in private practice? But this much may perhaps be said. It is very desirable that any stand that a hospital may take in this matter should be made on good grounds, since the law and the hospital are liable to take opposite views of the question. As the ultimate decision may rest with the law, a very important issue had better be left to lie than be fought unsuccessfully on a minor application of it.

---

### OPEN SPACES AND HOSPITAL EXTENSIONS.

The Birmingham General Hospital has asked the City Council to give up part of the old burial ground of St. Mary's Church in order that the hospital may be extended. A decision has been postponed until October, but the plan is likely to be opposed because the ground desired is laid out as an open space, and described as the only green spot in this corner of the city. The chairman of the Parks Committee, however, has declared in favour of the hospital's plan. He says its claim is paramount, and that the ground required is not a playground but a thoroughfare. The bodies resting in the old churchyard it is proposed to remove and re-bury at a cost of £20,000, but, because the neighbourhood is so crowded, the site for a hospital is far from ideal. We have said enough to show that the plan bristles with difficulties, and why any decision is advisably postponed. But the question has a larger importance. The difficulties of the General Hospital increase so steadily that the day is clearly coming when its future policy will have to be considered on statesmanlike lines. The advocates of its removal, on medical and financial grounds alike, are sure to make themselves heard, and we welcome this revival of a big question because there is a body of opinion in Birmingham which is far too ready to assume that the arguments in favour of removing urban hospitals to the outskirts cease to be valid in Birmingham itself. The real question is whether the hospital could afford to remove. If the answer at present is No, does not encroachment on the few open spaces in the heart of the city become doubly undesirable?

---

### OPPOSITE POLICIES AT BATH AND BIRMINGHAM.

Shortly before the Birmingham General Hospital asked the City Council to surrender its interest in St. Mary's Church and the upper part of the churchyard for an extension to the hospital, Lord Dawson of Penn was addressing a medical gathering at the Forbes Fraser Hospital, Bath. The subject of his address was the advantage of constructing a hospital outside a town, where ample ground and fresh air are available, and extensions can easily be made. Lord Dawson went on to advocate the pavilion style of building, and emphasised that the cost of a pavilion was one half that of a storeyed hospital. The single floor should be used, he said, until, say, 100 beds were reached; for hospitals of 150 to 200 beds two storeys. It was possible to build two-storeyed buildings of the same fabric as a pavilion, and the cost of construction would be little more. These arguments should be remembered at Birmingham, where the latest proposal will involve construction on an open space, the building of a new church to replace St. Mary's, the re-burial of the bodies removed, and a private Bill in Parliament. The hospital will have to find the money for all these if the scheme goes through, and thus a vast price will have to be paid for an extension on the very spot when it is of the most questionable advantage.

---

### BRADFORD CORPORATION AND THE INFIRMARY

Now that the Bradford City Council has twice referred back the sub-committee's recommendation to purchase the site and buildings of the Infirmary, the latest proposal is that the Corporation should make a grant of £45,000 towards the projected new hospital. The original plan is now out of favour, on the ground that the present infirmary buildings would be of no use to the Corporation unless they were demolished to provide an open space in Westgate, which is a congested district. On the other hand, though the annual grant of the proceeds of a penny rate for five years would give to the Infirmary £46,000 instead of the £100,000 hoped for by the Infirmary's committee, yet the committee would still be able to sell the present site and buildings, and thus presumably raise the sum total that is needed. If this recommendation be adopted, the Corporation will seek representation upon the Infirmary board of management during the period when the contributions are being made. A solution is badly needed to a question that threatens to become a thorn in the side of both the parties interested.

### SMALLER HOSPITALS AS TRAINING SCHOOLS.

Mr. John Barker, of Grimsby and District Hospital, who at the recent Hospital Conference raised the question of the recognition of smaller hospitals by the General Nursing Council, has since published the issue of his correspondence with the Council. On July 25 the Council resolved, in effect, that hospitals which, though sufficiently large, cannot be approved as complete training schools because of some deficiency of clinical material must associate themselves with another hospital or hospitals where the deficiency can be made good. The institutions, entitled "Associated Hospitals," which propose such arrangements will have to satisfy the Council that a complete training is thereby secured. The period of training must occupy three years and six months. This rule is being added to the scheme for the training of nurses, who will be admitted to the register after its provisions come into operation. The smaller hospitals thus have a means of supplying a want for which they are not responsible, and the nurses trained at them should not be at any disadvantage. These matters affect the smaller hospitals in many indirect but important respects, and their solution only depends upon the successful working out of the principle adopted by the Council.

### THE NEW DONCASTER INFIRMARY.

Now that the board of the Doncaster Infirmary has decided to adopt the Thorne Road site, a long controversy, we hope, is to be satisfactorily settled. Everyone agrees that a new infirmary is necessary to provide for the hospital needs of a district the development of the coalfield has created, but a section of the governors has favoured the site of the house bequeathed by the late Miss Beckett-Denison. These governors abstained from voting when the recent choice was made. On the other hand, the Thorne Road site has been acquired, and the representatives of the mining industry are in favour of building upon it. The opposition therefore has not taken part in this decision, and may perhaps make itself heard at a later stage. Probably their chance will come when the disposal of the present premises has to be decided, but great weight must attach to the attitude of the miners, since their needs are the crux of the problem. In the circumstances, then, we trust that unanimous councils will prevail, and the minority accept a decision already overdue that has been made only after careful thought.

### HOLIDAY PATIENTS AND COTTAGE HOSPITALS.

The experience of town hospitals in receiving patients, with but little support, from outlying districts is now being reversed. Cottage hospitals, especially those on the main roads leading from the towns, are now similarly situated. The reason is the increase, especially during the summer, of motor traffic. Every week end or public holiday brings a stream of cars from town to country, and accidents are proportionately frequent. The victims have often to be taken to the nearest hospital, which is frequently a cottage hospital close to the main road. The sufferers usually come from a distance and are bound to increase with the steady growth of the motor. Presumably many of them contribute. It is suggested, nevertheless, that, since the towns can hardly be made to contribute, the various automobile associations should be approached for subscriptions. What is the experience of most cottage hospitals in this matter ?

### THE FUTURE OF DUBLIN HOSPITALS.

The ultimate object of the endeavour to raise £100,000 for Sir Patrick Dun's Hospital is to establish a central institution incorporating Sir Patrick Dun's, the Adelaide, Meath, Mercer's and the Royal City of Dublin Hospitals. A great institution would be built, probably on the site of the first-named, because of its convenience alike for patients and doctors. The medical boards of all five hospitals are said to be in favour of the scheme, and, if the great sum be raised, it is believed that the hospital may secure a large grant from the Rockefeller Trust. Such grants are made only for research, and the scheme is designed to give to Dublin a hospital centre comparable to any in the world. There is thus much more than an isolated appeal in question, and it is not too much to say that the future of Dublin's hospitals and Dublin's medical education is in the balance.

### MR. CHESTERTON ON HOSPITAL APPEALS.

A series of luncheons held every day during a week is one of the methods adopted by the committee for raising the money. Mr. G. K. Chesterton, who was in Dublin for the Games, was one of the speakers at the opening luncheon. He said that a man had generally to be first knocked down by an omnibus to discover what work the hospitals do, since medical science, unlike other professions, could only advertise itself to those whom it relieved. He is not reported to have added that hospital appeals could only ensure success by organising a holocaust of wealthy victims, but his humour does explain why hospitals find it hard to raise money, since they have to appeal to those who hope never to feel the need of their assistance. It is characteristic that Mr. Chesterton's first hospital appeal should have been made, we believe, on behalf of an Irish hospital. The Dublin hospitals are in a very serious financial plight, and the international throng in the city for the Games and the Horse Show needed some telling reminder of their claim for help. In the joint celebrations we hope that this claim will have been heard.

